# Aging Unequally: Functional Age Disparities Between Developmental and Non-Developmental Disabilities

**DOI:** 10.3390/healthcare13192412

**Published:** 2025-09-24

**Authors:** Ji Ung Jeong

**Affiliations:** Department of Disability Studies, Daegu University, Gyeongsan-si 38453, Republic of Korea; jiungjeong987@gmail.com

**Keywords:** developmental disabilities, accelerated aging, functional age, health disparities, functional limitation

## Abstract

**Background**: Adults with developmental disabilities often experience accelerated aging, but the magnitude of this phenomenon is not well quantified. This study aimed to measure the disparity in functional ability and chronic illness prevalence between adults with developmental and other disabilities. **Methods**: A “functional age” was calculated for adults with developmental disabilities. This metric, designed as a statistical index of disparity, was derived from normative regression models of ADL and IADL based on a reference group of adults with other disabilities. **Results**: A profound gap was found between chronological and functional age. On average, a 44-year-old individual with a developmental disability exhibited a level of functional limitation equivalent to a person over 100 years older in the reference population for both ADL and IADL (*p* < 0.001). **Conclusions**: Accelerated aging in this population manifests as a severe, early onset functional disadvantage rather than an elevated burden of general chronic disease. Policies should shift toward function-based, not age-based, models of care to address these lifelong support needs.

## 1. Introduction

A central paradox of modern healthcare is that increased longevity does not always equate to an extended period of health [1,2]. This is particularly true for individuals with developmental disabilities—a broad category encompassing intellectual disabilities, autism spectrum disorder, and cerebral palsy—who are now living longer than ever before [3,4]. This demographic shift has created an aging population with a unique and complex health profile, characterized by the long-observed clinical phenomenon of “accelerated aging” [5]. This phenomenon describes the earlier onset of age-related health conditions, premature functional decline, and mortality compared to the general population [6,7]. Research shows that many individuals with developmental disabilities experience physical changes and mobility limitations in their 40s and 50s, mirroring patterns typically seen in neurotypical adults decades older [8]. This apparent disconnect between chronological and biological age suggests that standard age-based models of care are inadequate, highlighting a critical need to understand the specific nature and timing of the aging process in this group [9].

The concept of accelerated aging is supported by extensive, multidimensional evidence that spans biological, clinical, and functional domains. Biologically, the phenomenon is often attributed to factors inherent in certain conditions—such as increased oxidative stress and chronic low-grade inflammation in Down syndrome—combined with a higher prevalence of lifestyle risks like physical inactivity and obesity across the broader developmental disability population [10,11,12]. These underlying mechanisms are believed to hasten the onset of cellular senescence and contribute to a state of increased physiological vulnerability [13].

Clinically, this leads to an earlier emergence of conditions typically associated with old age. For individuals with Down syndrome, the genetic overexpression of amyloid precursor protein means the neuropathological hallmarks of Alzheimer’s disease are nearly universal by age 40, with corresponding declines in adaptive functioning occurring decades earlier than in the general population [14]. Beyond this specific example, adults with intellectual and developmental disabilities (IDD) as a whole show a higher burden of multimorbidity, including an earlier onset of osteoporosis, sensory impairments, and cardiovascular disease [15,16].

This decline is typically assessed through limitations in Activities of Daily Living (ADLs), which refer to basic self-care activities like eating and bathing, and Instrumental Activities of Daily Living (IADLs), which encompass more complex tasks necessary for independent community living, such as managing finances or shopping [17,18]. Hilgenkamp et al. [19] reported that adults with intellectual disabilities exhibit significantly impaired performance on ADL and IADL by their 50s. Similarly, adults with cerebral palsy often encounter age-related declines in strength, increased pain, and new mobility limitations—so-called “secondary health conditions”—at a much younger age than their peers, often leading to a loss of independent ambulation in early to middle adulthood [20,21]. This evidence collectively paints a consistent picture of a population facing the functional and health challenges of old age while still in their middle years.

These physiological declines are deeply intertwined with social determinants of health that create a “cascade of disparities” [5]. As individuals with developmental disabilities age, they are more likely to experience poverty, social isolation, and inadequate healthcare, which together heighten physiological vulnerability [22]. This is often exacerbated by “diagnostic overshadowing,” a pervasive cognitive bias wherein clinicians mistakenly attribute physical symptoms of a co-occurring condition to the primary developmental disability, leading to missed or delayed diagnoses [23,24]. Although mortality rates remain higher in this population, those surviving into midlife often face levels of functional impairment far greater than would normally be expected for their chronological age [11,25]. This phenomenon has profound implications, impacting everything from individual quality of life and caregiver burden to the design of long-term support services and public health policy [26,27].

Yet a comprehensive, population-level understanding of this disparity remains limited by methodological challenges that restrict accurate assessment of accelerated aging. Much of the research to date is based on narrowly defined clinical cohorts or restricted to specific diagnostic groups, such as Down syndrome or intellectual disability. While such studies have provided foundational insights—such as the early emergence of Alzheimer’s pathology in individuals with Down syndrome [28], or midlife declines in ADL/IADL functioning in those with intellectual disabilities [19]—their findings are often not generalizable to the broader and more diverse developmental disability population. This is particularly problematic given the variability in functional status, comorbidity profiles, and support needs across different subgroups [5,29]. In addition, comparisons to the general population—though valuable for identifying broad disparities—often fail to disentangle the effects of early onset, lifelong disability from those of later-acquired impairments. Such approaches risk obscuring critical differences in developmental trajectories, health risks, and service needs [16,24].

To disentangle these factors, a more insightful comparison—pitting adults with developmental disabilities against their peers with other, later-onset disabilities—is necessary. This comparison is crucial for isolating the unique impact of a condition present from early life, a principle central to the life course health development model, which posits that the timing and duration of health exposures fundamentally shape long-term outcomes [30]. By using adults with other disabilities as a reference, it becomes possible to control for the general experience of disability and focus specifically on the “accelerated” component attributable to developmental conditions. Yet, such direct comparisons, especially using quantitative functional metrics, are notably uncommon in large-scale research. Consequently, a clear, quantitative assessment of this specific “health gap” using nationally representative data is needed to move beyond the limitations of clinical observation and inform evidence-based practice at a population level [3].

The present study addresses this gap by utilizing nationally representative data and a functional aging framework that enables direct, empirically grounded comparisons. By calculating “functional age” based on normative models of aging from a reference group, this approach moves beyond descriptive comparisons to offer a quantifiable measure of health disparity. In doing so, this study provides the kind of rigorous, population-level evidence needed to inform inclusive health policy, targeted service provision. Therefore, the primary research aims are to determine: whether adults with developmental disabilities exhibit functional limitations in ADL and IADL that are disproportionate to their chronological age; and whether this pattern of accelerated aging applies equally to the prevalence of chronic illness. This study proposes a shift toward function-based aging metrics as a more equitable foundation for care planning, health screening, and resource allocation. In doing so, it aims to contribute to a more just and evidence-informed understanding of aging in the developmental disability population.

## 2. Method

### 2.1. Data and Sample

This study utilizes data from the 18th wave of the Korean Welfare Panel Study (KOWEPS), a nationally representative longitudinal survey conducted in 2023 [31]. KOWEPS is jointly managed by the Korea Institute for Health and Social Affairs and Seoul National University since 2006. The survey is designed to track the economic, social, and health conditions of households and individuals across South Korea, with a particular emphasis on low-income and vulnerable populations. The analysis leverages the main survey data in conjunction with the supplemental survey on persons with disabilities, which contains detailed information on disability characteristics, health, and functional status. The target population of KOWEPS includes all private households in South Korea as of 2022, excluding special facilities and island regions. The sample was drawn using a two-stage stratified sampling method based on the 2005 Korean Population and Housing Census. From this pool, 7000 households (3500 general and 3500 low-income) were chosen to ensure balanced representation across income levels and enhance the statistical efficiency of the panel.

To quantify the phenomenon of accelerated aging, this study employed a two-part sampling strategy to compare the functional health status of adults with developmental disabilities to their peers. As this study utilized a pre-existing, nationally representative dataset, an a priori sample size calculation was not performed. The eligibility criteria for the sample included all adults with a registered disability in the KOWEPS dataset. The reference group, used to build the normative aging models for ADL and IADL, consisted of all 1364 individuals in the dataset with non-developmental disabilities. The focal group for the primary analysis comprised all individuals with a registered developmental disability (*N* = 93). To ensure analytical consistency, one case with missing data on key variables was excluded, making the final analytical sample for the paired-samples *t*-tests 92 individuals from this focal group.

This study was a secondary analysis of cross-sectional data using publicly available, de-identified data from the KOWEPS. Therefore, it was exempt from institutional review board approval. The original KOWEPS survey was conducted in accordance with the Declaration of Helsinki, and the protocol was approved by the Institutional Review Board of the Korea Institute for Health and Social Affairs.

### 2.2. Measures

The first dependent variable, ADL, measured functional limitation using a 12-item scale assessing an individual’s ability to perform basic self-care tasks such as dressing, bathing, and eating. Each item was rated on a 3-point scale from 1 (“Completely Independent”) to 3 (“Needs Complete Assistance”). The responses were summed to create a total ADL score ranging from 12 to 36, where a higher score indicates a greater level of functional dependence. The second dependent variable, IADL, assessed the capacity for more complex independent living tasks like managing finances or preparing meals via a 10-item scale. Responses of “Not Applicable” were treated as missing data, while remaining items were rated on the same 3-point scale as the ADL. A total IADL score was computed by summing the valid responses, with a higher score signifying greater difficulty with instrumental tasks. The third dependent variable, Presence of Chronic Illness, measured general health status using an item assessing the duration of treatment for a chronic condition. This variable was treated as an ordinal outcome with four levels: “No Illness,” “Treatment for less than 3 months,” “Treatment for 3–6 months,” and “Treatment for 6 months or more.”

The primary independent variable was Disability Type, a dichotomous variable where individuals with intellectual or autism spectrum disorders were coded as 1 (“Developmental Disability”), and individuals with all other disability types served as the reference group (coded as 0).

To isolate the relationship between disability type, age, and health outcomes, several key sociodemographic and economic variables were included as controls. Age was included as a continuous variable. Sociodemographic controls included Sex (1 = Male, 0 = Female), Education Level (an ordinal scale), and Marital Status (1 = Partnered, 0 = Other). Economic status was controlled for using the natural logarithm of the household’s annual disposable income. Finally, a dummy variable for Disability Severity (1 = Severe, 0 = Mild) was included to account for baseline differences in health status.

### 2.3. Analytical Strategy

A multi-stage analytical strategy was employed, tailored to the measurement level of each dependent variable.

#### 2.3.1. Analysis of Functional Limitations (ADL and IADL)

For the continuous outcome variables (ADL and IADL), a two-stage “functional age” model was used. This approach was constructed to create a quantifiable index of health disparity, following the precedent of similar “biological age” metrics in other health fields, such as “lung age” in pulmonology [32] and “brain age” in neuroimaging [33]. The first stage involved establishing a covariate-adjusted normative aging trajectory by estimating a multiple linear regression model on the reference population of individuals with non-developmental disabilities. The general form of this normative model was:

Predicted Score_i_ = β_0_ + β_1_ (Age_i_) + β_2_ (Sex_i_) + … + β_n_ (Covariate_ni_) + ε_i_

In the second stage, a “functional age” was calculated for each individual in the developmental disability group. This was achieved by inverting the regression equation and using it to solve for the age at which a person in the reference group would be expected to have the same functional score, after accounting for all other covariates. The general formula for this calculation was:

Functional Age_i_ = ([Actual Score_i_ − Σ(β_n_ × Covariate_Value_ni_)] − β_0_)/β_1_

Finally, a paired-samples *t*-test was conducted to determine if there was a statistically significant difference between the chronological age and the calculated functional age for the developmental disability group.

#### 2.3.2. Analysis of Chronic Illness Prevalence

As the “Presence of Chronic Illness” variable is ordinal, a different analytical approach was required. First, an ordinal logistic regression model was estimated to assess the relationship between the predictor variables and the likelihood of being in a higher category of chronic illness duration. The general form of the underlying linear model is:

Logit(P(Y ≤ j)) = Threshold_j_ − (β_1_ (DisabilityType_i_) + β_2_ (Age_i_) + … + β_n_ (Covariate_ni_))

Second, to illustrate the practical magnitude of the effect of disability type, the coefficients from this model were used to calculate and compare the predicted probabilities of having a chronic illness for a “typical” individual profile (e.g., based on the sample mean/mode for covariates), once for each disability group. This comparison serves to quantify any observed health gap in an intuitive, probabilistic framework.

All analyses were conducted using SPSS version 28, with statistical significance set at *p* < 0.05.

#### 2.3.3. Patient and Public Involvement

It was not possible to involve patients or members of the public in the design, conduct, or reporting of this research. This study is a secondary analysis of a pre-existing, de-identified public-use dataset. As such, the research questions and analytical strategy were formulated based on the available data, and there was no direct contact with the study participants. The findings of this study will be disseminated through academic publication to reach clinicians, policymakers, and advocacy groups working with this population.

## 3. Results

### 3.1. Descriptive Characteristics of the Sample

The descriptive statistics of the study sample are presented in Table 1. The final sample comprised 1457 individuals with disabilities, of whom 93 (6.4%) were identified as having a developmental disability and 1364 (93.6%) had other types of disabilities.

An independent samples *t*-test revealed significant demographic and socioeconomic differences between the two groups. The developmental disability group was substantially younger on average (M = 44.17, SD = 14.63) compared to the group with other disabilities (M = 72.76, SD = 12.52), a difference that was statistically significant, *t*(101.41) = 18.40, *p* < 0.001. Conversely, the average annual household disposable income was significantly higher for the developmental disability group (M = 44.68 million KRW) than for the other disabilities group (M = 31.42 million KRW), *t*(103.94) = −4.06, *p* < 0.001. Regarding the dependent variables, there was no statistically significant difference in the level of ADL limitation between the developmental disability group (M = 13.75) and the other disabilities group (M = 13.03), *t*(102.74) = −1.82, *p* = 0.072. However, a significant difference was observed for IADL limitations. The developmental disability group reported a significantly higher mean IADL limitation score (M = 16.99, SD = 5.98) than the other disabilities group (M = 12.62, SD = 4.71), indicating greater difficulty with instrumental daily tasks, *t*(99.46) = −6.86, *p* < 0.001.

Further analysis using chi-square tests showed significant differences in the distribution of categorical variables between the two groups (see Table 1). The proportion of males was significantly higher in the developmental disability group (64.5%) compared to the other disabilities group (46.9%), χ^2^(1, *N* = 1457) = 10.80, *p* = 0.001. Significant differences were also found in educational attainment, χ^2^(4, *N* = 1457) = 43.51, *p* < 0.001. A notable concentration of individuals with developmental disabilities was observed at the high school level (50.5%), whereas the other disabilities group was most concentrated at the elementary school level (37.3%). Marital status also differed significantly between the groups, χ^2^(1, *N* = 1457) = 57.26, *p* < 0.001. Most individuals with developmental disabilities were single/unpartnered (87.1%), while the other disabilities group was more evenly split between being partnered (53.4%) and unpartnered (46.6%). A stark and highly significant difference was found in disability severity, χ^2^(1, *N* = 1457) = 186.71, *p* < 0.001. All individuals (100.0%) in the developmental disability group were classified as having a severe disability, compared to only 30.3% in the other disabilities group. Finally, the prevalence of chronic illness differed significantly, χ^2^(3, *N* = 1457) = 69.59, *p* < 0.001. A larger proportion of the other disabilities group reported long-term treatment (6+ months) for a chronic condition (89.4%) compared to the developmental disability group (62.4%). Conversely, a higher percentage of the developmental disability group reported having no chronic illness (35.5%) compared to the other group (8.6%).

### 3.2. Functional Age Analysis for ADL

To quantify the functional health disparity suggested by the descriptive data, the “functional age” methodology was employed. This analysis first established a normative aging trajectory for ADL limitations and then used this benchmark to evaluate the developmental disability group.

#### 3.2.1. Normative Model of ADL Limitation

A multiple linear regression was estimated using the reference group of 1364 individuals with non-developmental disabilities. The model predicted the ADL limitation score from chronological age and a set of sociodemographic and economic covariates. The results are presented in Table 2.

The overall model was statistically significant, F(6, 1244) = 19.45, *p* < 0.001, and explained 8.6% of the variance in ADL scores (*R*^2^ = 0.086). In this model, both chronological age (B = 0.048, *p* < 0.001) and disability severity (B = 2.018, *p* < 0.001) were significant positive predictors of ADL limitation, indicating that greater age and having a severe disability were associated with worse functional outcomes. Household income also emerged as a significant predictor (B = 0.530, *p* = 0.001).

#### 3.2.2. Comparison of Chronological and Functional Age

Using the coefficients from the normative model, a functional age was calculated for each of the 92 individuals in the developmental disability group. A paired-samples *t*-test was then conducted to compare their mean chronological age with their mean calculated functional age.

The results, shown in Table 3, revealed a profound and statistically significant disparity. The mean chronological age of the developmental disability group was 44.25 years (SD = 14.69). In stark contrast, their mean functional age based on ADL limitations was 188.77 years (SD = 76.94). This difference of −144.52 years was highly significant, *t*(91) = −17.11, *p* < 0.001.

This finding indicates that an average 44-year-old with a developmental disability exhibits a level of functional limitation in basic daily activities that is comparable to a person aged 188 years in the reference population. While the calculated age is outside the human lifespan, it serves as a powerful metric illustrating the extreme severity of the functional health gap between the two groups.

### 3.3. Functional Age Analysis for IADL

To determine if the pattern of accelerated aging extends to more complex daily tasks, the same functional age analysis was repeated for IADL.

#### 3.3.1. Normative Model of IADL Limitation

A multiple linear regression was estimated on the reference group to establish a normative trajectory for IADL limitations. The results of this model are detailed in Table 4.

The overall model was highly significant, F(6, 1244) = 38.97, *p* < 0.001, explaining 15.8% of the variance in IADL scores (*R*^2^ = 0.158). Similarly to the ADL model, both chronological age (B = 0.107, *p* < 0.001) and disability severity (B = 3.665, *p* < 0.001) were strong, positive predictors of IADL limitation. Household income was also a significant predictor (B = 0.443, *p* = 0.043). The coefficients from this model were used to derive the normative formula for calculating IADL-based functional age.

#### 3.3.2. Comparison of Chronological and Functional Age

The IADL-based functional age was subsequently calculated for each of the 92 individuals in the developmental disability group. A paired-samples *t*-test was then performed to compare their chronological age with their newly calculated functional age.

As shown in Table 5, the results confirmed a significant and substantial gap, consistent with the ADL findings. The mean chronological age of the group was 44.25 years (SD = 14.69), while their mean functional age based on IADL limitations was 155.55 years (SD = 55.88). The mean difference of −111.30 years was highly statistically significant, *t*(91) = −17.86, *p* < 0.001.

This result demonstrates that the functional disadvantage extends robustly to instrumental tasks. On average, a 44-year-old with a developmental disability shows a level of functioning in complex daily activities comparable to that of a 155-year-old with another type of disability. This provides a second, powerful line of evidence supporting the concept of accelerated aging.

### 3.4. Analysis of Chronic Illness Prevalence

Finally, the analysis shifted from functional health to general health status, as measured by the presence and duration of a chronic illness. Given the ordinal nature of this outcome, the analytical approach differed from the functional age model. An ordinal logistic regression was first employed to assess the relationship between the predictor variables and the likelihood of reporting a longer duration of chronic illness.

#### 3.4.1. Ordinal Logistic Regression Model

The results of the ordinal logistic regression are presented in Table 6. The overall model was statistically significant, indicating that the set of predictors reliably distinguished between levels of chronic illness (χ^2^(8) = 25.40, *p* = 0.001). Chronological age (*p* = 0.004), education level (*p* = 0.008), and household income (*p* = 0.021) were all significant predictors.

Crucially, the main effect for Disability Type was not statistically significant (*p* = 0.617). This finding suggests that, after controlling for age, severity, and other socioeconomic factors, there is no significant difference in the underlying likelihood of having a long-term chronic illness between the developmental disability group and the reference group.

#### 3.4.2. Comparison of Predicted Probabilities

To illustrate the practical implications of this non-significant finding, predicted probabilities were calculated based on the model for a representative individual: a 50-year-old male with a severe disability, a high school education, and an average household income.

As shown in Table 7, the results confirm the lack of a substantial health gap for this outcome. For an individual with a developmental disability, the predicted probability of having a chronic illness lasting six months or more was 63.5%. For an otherwise identical individual with a non-developmental disability, the corresponding probability was 58.5%. This small, 5-percentage-point difference aligns with the statistical non-significance of the disability type variable in the regression model.

Thus, while the analyses for ADL and IADL provide strong evidence for accelerated functional aging, this pattern does not extend to the general prevalence of long-term chronic illness.

## 4. Discussion

### 4.1. Interpretation of Key Findings

The most striking result of this study is the massive gap in functional age between adults with developmental disabilities and the normative trajectory derived from individuals with other types of disabilities. While the average chronological age of the developmental disability group was just 44.25 years, their functional age based on ADL limitations was 188.77 years, and 155.55 years based on IADL limitations. Both differences were highly significant (ADL: *t*(91) = −17.11, *p* < 0.001; IADL: *t*(91) = −17.86, *p* < 0.001). This indicates that a middle-aged adult with a developmental disability exhibits a level of functional limitation comparable to someone over 100 years older in the reference population—a finding that powerfully operationalizes the concept of accelerated functional aging [19]. It is critical to interpret this “functional age” not as a literal biological prediction but as a statistical index of disparity. The extreme values serve to quantify the immense scale of the functional health gap when mapped against a normative trajectory, illustrating a severe, early onset functional disadvantage.

Notably, this disparity is not simply a product of disease accumulation in old age. The descriptive data revealed that, although individuals with developmental disabilities were significantly younger on average than their peers (*p* < 0.001), they nonetheless reported significantly worse IADL scores (*p* < 0.001) and similar, albeit not statistically different, ADL limitations (*p* = 0.072). When age, income, and other confounders were controlled for in the functional age model, these underlying disparities became even more pronounced, suggesting a persistent and severe functional disadvantage that begins early in the life course.

These findings align with extensive clinical research indicating that people with developmental disabilities often experience early and persistent functional impairment that differs from typical aging patterns [16,19,20]. Down syndrome provides a key example, where the manifestation of functional regression and neurodegenerative symptoms can commence as early as the third decade of life [12,34,35]. These impairments are often lifelong and may be compounded by a “cycle of disadvantage,” including limited access to habilitative services, social exclusion, and inadequate aging-in-place supports, which further exacerbates health inequities [24,36,37]. Building upon the argument that midlife is a critical period for study [38], this research demonstrates that for adults with developmental disabilities, it is a period in which significant health inequities are already firmly established.

In contrast to the profound functional limitations, the prevalence of chronic illness did not differ significantly between groups after controlling for age and other covariates (*p* = 0.617). The predicted probability analysis confirmed this, showing only a small and statistically non-significant difference in the likelihood of reporting long-term illness between the groups. At first glance, this might suggest that individuals with developmental disabilities do not bear an elevated general health burden. However, this interpretation warrants significant caution, as it may reflect systemic healthcare inequities rather than true health parity.

A substantial body of literature has highlighted that diagnostic overshadowing—where clinicians attribute physical symptoms to an individual’s primary disability rather than investigating for co-occurring conditions—can lead to the underdiagnosis of treatable illnesses in this population [5,23]. This is a well-documented barrier that can suppress reported prevalence rates of common conditions like hypertension, diabetes, and gastrointestinal disorders [39]. Additionally, communication challenges, limited health literacy, and systemic barriers to preventive care further distort the accurate reporting and diagnosis of chronic conditions [26,40].

Moreover, the demographic differences between the groups must be considered. The developmental disability group was, on average, nearly 30 years younger than the comparison group. Therefore, the very fact that they had statistically comparable levels of long-term illness, despite this large age gap, could itself be interpreted as evidence of an earlier onset of chronic conditions that remains statistically undetected due to the limitations of cross-sectional data or the competing risk of premature mortality. These findings reinforce the idea that accelerated aging appears clearly in functional limitations but not consistently across all areas of health. The functional disadvantage appears early and is severe, likely reflecting both inherent developmental factors and cumulative disadvantage, while the lack of disparity in chronic illness prevalence may be an artifact of underdiagnosis and other systemic barriers.

### 4.2. Implications for Policy

The findings of this study, particularly the profound gap between chronological and functional age highlight the need for the prudent reform and targeted strengthening of existing support structures to enhance their efficiency. The results underscore three key policy applications:

First, the findings challenge the efficacy of using chronological age as a primary gateway for accessing long-term services and supports. A shift to a function-based eligibility model is not a call for expanded entitlement, but a fiscally responsible measure to ensure that public resources are directed with precision to individuals with demonstrated need. By aligning support with functional status rather than an arbitrary age, policymakers can better steward taxpayer funds, prevent waste, and ensure that interventions have the greatest possible impact.

Second, the early entrenchment of severe functional limitations calls for proactive, integrated support models that begin well before the conventional threshold of old age. This approach also aligns with principles of individual empowerment, allowing for more tailored, consumer-directed support packages that can adapt to changing needs over the life course [41]. Such systems are essential for upholding the rights of persons with disabilities, as outlined in the UN Convention on the Rights of Persons with Disabilities (CRPD), particularly Article 28 (right to an adequate standard of living and social protection).

Finally, the potential for underdiagnosis of chronic illness points to a critical need to improve diagnostic practices within the existing healthcare system. Diagnostic overshadowing leads to poor health outcomes and often results in more costly emergency interventions down the line. A prudent, low-cost strategy to address this is to promote the widespread adoption of evidence-based clinical guidelines and enhance provider education for the primary care of adults with developmental disabilities [39]. Investing in the capacity of the current healthcare infrastructure to provide competent care—thereby fulfilling Article 25 of the CRPD (right to health)—is a more sustainable solution than creating separate, parallel systems. By improving diagnostic accuracy, the existing system can operate more effectively, improve long-term health, and reduce the future fiscal burden associated with managing unaddressed chronic conditions.

### 4.3. Limitations and Future Research

While this study provides important insights, several limitations must be acknowledged. First, the analysis is based on cross-sectional data from a single wave of the KOWEPS. Although the functional age methodology provides a robust snapshot, longitudinal data tracking the same individuals over time would be necessary to definitively measure the rate of decline and confirm the trajectories suggested here. Second, the measure of chronic illness was based on self-report of treatment duration, which may not capture undiagnosed conditions. The findings regarding chronic illness should therefore be interpreted with caution, as they may reflect disparities in healthcare access as much as true prevalence. Third, the developmental disability category is heterogeneous, encompassing a wide range of conditions and support needs. The current analysis, while informative, could not disaggregate between different types of developmental disabilities (e.g., intellectual disability vs. autism spectrum disorder) due to sample size constraints. Fourth, a formal sensitivity analysis was not performed to test the robustness of the model estimates. Collectively, these limitations, including the small focal group size and potential for survivorship bias, mean the findings should be viewed as indicative of a profound disparity rather than as definitive, generalizable estimates.

Future research should prioritize longitudinal analysis to track health and functional trajectories over time. Additionally, studies incorporating direct health measures (e.g., biomarkers) alongside self-reported data could help disentangle the effects of inherent functional limitations from the impact of undiagnosed chronic disease, providing a clearer picture of the mechanisms driving the health disparities observed in this study. Lastly, the finding that chronic illness prevalence is not higher among people with developmental disabilities deserves further investigation—especially into diagnostic access, survivorship bias, and cause-of-death data.

## 5. Conclusions

This study set out to examine whether adults with developmental disabilities exhibit signs of accelerated aging compared to individuals with other types of disabilities. The analysis revealed a pronounced and statistically significant disparity in functional ability, but not in general morbidity. Adults with developmental disabilities demonstrated functional profiles consistent with individuals several decades older, despite their relatively young chronological age. In contrast, the study did not find significant differences in the prevalence or duration of chronic illness between the developmental disability group and the reference group. This divergence suggests that the aging process in individuals with developmental disabilities may follow a unique path—marked more by a severe disadvantage in daily functioning than by an elevated burden of traditional chronic diseases.

Taken together, these findings emphasize the need to reconceptualize aging in the context of developmental disability. The results support a model of early onset functional decline, showing that limitations arise earlier and progress more severely, warranting earlier intervention, proactive support planning, and age-independent access to services. Standard age-based models of care and policy may be insufficient or even exclusionary when applied uniformly. This study reframes the concept of accelerated aging from a simple measure of a faster rate of decline to a more complex picture of early and severe functional disadvantage.

## Figures and Tables

**Table 1:** Descriptive Characteristics of the Study Sample by Disability Type.

Characteristic	Developmental Disability (*n* = 93)	Other Disabilities (*n* = 1364)	Test Statistic	*p*-Value
**Continuous Variables** (*Mean* ± *SD*)			***t*-test**	
Age (years)	44.17 ± 14.63	72.76 ± 12.52	18.40	**<0.001**
Household Income (10,000 KRW/year)	4467.86 ± 3057.49	3141.92 ± 2940.20	−4.06	**<0.001**
ADL Limitation Score	13.75 ± 3.69	13.03 ± 3.42	−1.82	0.072
IADL Limitation Score	16.99 ± 5.98	12.62 ± 4.71	−6.86	**<0.001**
**Categorical Variables *n* (%)**			**χ^2^-test**	
**Sex**			10.80	**0.001**
Female	33 (35.5)	724 (53.1)		
Male	60 (64.5)	640 (46.9)		
**Education Level**			43.51	**<0.001**
No Schooling	5 (5.4)	197 (14.4)		
Elementary School	23 (24.7)	509 (37.3)		
Middle School	10 (10.8)	244 (17.9)		
High School	47 (50.5)	291 (21.3)		
University or Higher	8 (8.6)	123 (9.0)		
**Marital Status**			57.26	**<0.001**
Unpartnered (Single, Divorced, etc.)	81 (87.1)	635 (46.6)		
Partnered (Married, etc.)	12 (12.9)	729 (53.4)		
**Disability Severity**			186.71	**<0.001**
Mild	0 (0.0)	951 (69.7)		
Severe	93 (100.0)	413 (30.3)		
**Chronic Illness Status**			69.59	**<0.001**
No Illness	33 (35.5)	117 (8.6)		
<3 months of treatment	2 (2.2)	18 (1.3)		
3–6 months of treatment	0 (0.0)	10 (0.7)		
6+ months of treatment	58 (62.4)	1219 (89.4)		
**Total Sample**	**93 (100.0)**	**1364 (100.0)**		

**Table 2:** Regression Model for Normative ADL Limitation Trajectory (Reference Group Only).

Predictor	B	Std. Error	β	*t*	*p*-Value
(Constant)	4.689	1.637		2.864	0.004
Age (years)	0.048	0.010	0.171	4.913	<0.001
Sex (Male)	0.005	0.205	0.001	0.024	0.980
Education Level	0.035	0.101	0.012	0.344	0.731
Marital Status (Partnered)	0.053	0.218	0.008	0.242	0.809
Household Income (ln)	0.530	0.165	0.109	3.208	0.001
Disability Severity (Severe)	2.018	0.206	0.272	9.799	<0.001

Note: Dependent Variable = ADL Limitation Score. *N* = 1251. *R*^2^ = 0.086.

**Table 3:** Paired-Samples *t*-Test of Chronological Age vs. ADL-Based Functional Age (Developmental Disability Group).

Variable	Mean	Std. Deviation	*N*	*t*-Statistic	*df*	*p*-Value (2-Tailed)
Chronological Age	44.25	14.69	92			
Functional Age (ADL)	188.77	76.94	92	−17.11	91	<0.001
Mean Difference	−144.52	80.10	92			

**Table 4:** Regression Model for Normative IADL Limitation Trajectory (Reference Group Only).

Predictor	B	Std. Error	β	*t*	*p*-Value
(Constant)	0.345	2.167		0.159	0.874
Age (years)	0.107	0.013	0.278	8.313	<0.001
Sex (Male)	0.142	0.271	0.015	0.525	0.600
Education Level	−0.004	0.134	−0.001	−0.032	0.974
Marital Status (Partnered)	−0.263	0.289	−0.028	−0.908	0.364
Household Income (ln)	0.443	0.219	0.066	2.025	0.043
Disability Severity (Severe)	3.665	0.273	0.359	13.443	<0.001

Note: Dependent Variable = IADL Limitation Score. *N* = 1251. *R*^2^ = 0.158.

**Table 5:** Paired-Samples *t*-Test of Chronological Age vs. IADL-Based Functional Age (Developmental Disability Group).

Variable	Mean	Std. Deviation	*N*	*t*-Statistic	*df*	*p*-Value (2-Tailed)
Chronological Age	44.25	14.69	92			
Functional Age (IADL)	155.55	55.88	92	−17.86	91	<0.001
Mean Difference	−111.30	59.78	92			

**Table 6:** Ordinal Logistic Regression Model Predicting Duration of Chronic Illness.

Predictor	Estimate (B)	Std. Error	Wald	*df*	*p*-Value
Threshold [Illness = 0]	−4.396	2.231	3.882	1	0.049
Threshold [Illness = 1]	−4.271	2.230	3.667	1	0.055
Threshold [Illness = 2]	−4.253	2.230	3.638	1	0.056
Location					
Age (centered)	0.063	0.022	8.297	1	0.004
Education Level	0.443	0.167	7.026	1	0.008
Household Income (ln)	−0.588	0.254	5.359	1	0.021
Disability Type (Non-Dev)	−0.210	0.420	0.249	1	0.617
Sex (Female)	0.280	0.280	1.002	1	0.317
Marital Status (Unpartnered)	−0.347	0.369	0.882	1	0.348
Disability Severity (Mild)	−0.325	0.339	0.920	1	0.337

Note: *N* = 283. Model −2 Log Likelihood = 395.156. Nagelkerke *R*^2^ = 0.111.

**Table 7:** Predicted Probability of Chronic Illness Status by Disability Type.

Chronic Illness Status	Developmental Disability	Other Disabilities
No Illness	33.3%	38.1%
<3 months of treatment	2.8%	3.0%
3–6 months of treatment	0.4%	0.4%
6+ months of treatment	63.5%	58.5%

Note: Probabilities calculated for a 50-year-old male with severe disability, average income, and high school education.

## Data Availability

The data analyzed in this study are available from the Korean Welfare Panel Study (KOWEPS) data repository. Access can be requested at: https://www.koweps.re.kr:442/login.do (accessed on 6 September 2024).

## References

[1] Garmany A., Yamada S., Terzic A. (2021). Longevity leap: Mind the healthspan gap. NPJ Regen. Med..

[2] Hansen M., Kennedy B.K. (2016). Does longer lifespan mean longer healthspan?. Trends Cell Biol..

[3] Havercamp S.M., Scott H.M. (2015). National health surveillance of adults with disabilities, parents with disabilities, and their children. Disabil. Health J..

[4] Torr J., Strydom A., Patti P., Jokinen N. (2010). Aging in Down syndrome: Morbidity and mortality. J. Policy Pract. Intellect. Disabil..

[5] Krahn G.L., Hammond L., Turner A. (2006). A cascade of disparities: Health and health care access for people with intellectual disabilities. Ment. Retard. Dev. Disabil. Res. Rev..

[6] van Heumen L., Munly K., Heyn P. (2021). Aging with intellectual and developmental disabilities: A critical examination of supports for healthy aging. Innov. Aging.

[7] McCarron M., Swinburne J., Burke E., McGlinchey E., Mulryan N., Andrews V. (2013). Growing Older with an Intellectual Disability in Ireland 2013: First Results from the Intellectual Disability Supplement to the Irish Longitudinal Study on Ageing.

[8] Stephens M.M., Herge E.A., Rene R., Busby-Whitehead J., Durso S.C., Arenson C., Elon R., Palmer M.H., Reichel W. (2022). Aging in adults with intellectual disabilities and severe and persistent mental illness. Reichel’s Care of the Elderly: Clinical Aspects of Aging.

[9] Santos F.H., Zurek J., Janicki M.P. (2022). Efficacy of healthy aging interventions for adults with intellectual and developmental disabilities: A systematic review. Gerontologist.

[10] Rimmer J.H., Yamaki K. (2006). Obesity and intellectual disability. Ment. Retard. Dev. Disabil. Res. Rev..

[11] Tyrer F., Smith L.K., McGrother C.W. (2007). Mortality in adults with moderate to profound intellectual disability: A population-based study. J. Intellect. Disabil. Res..

[12] Lott I.T., Head E. (2019). Dementia in Down syndrome: Unique insights for Alzheimer disease research. Nat. Rev. Neurol..

[13] Widerström-Noga E., Finlayson M.L. (2010). Aging with a disability: Physical impairment, pain, and fatigue. Phys. Med. Rehabil. Clin. N. Am..

[14] Fortea J., Zaman S.H., Hartley S., Rafii M.S., Head E., Carmona-Iragui M. (2021). Alzheimer’s disease associated with Down syndrome: A genetic form of dementia. Lancet Neurol..

[15] O’Leary L., Cooper S.A., Hughes-McCormack L. (2018). Early death and causes of death of people with intellectual disabilities: A systematic review. J. Appl. Res. Intellect. Disabil..

[16] Hermans H., Evenhuis H.M. (2014). Multimorbidity in older adults with intellectual disabilities. Res. Dev. Disabil..

[17] Katz S., Ford A.B., Moskowitz R.W., Jackson B.A., Jaffe M.W. (1963). Studies of illness in the aged. The index of ADL: A standardized measure of biological and psychosocial function. JAMA.

[18] Lawton M.P., Brody E.M. (1969). Assessment of older people: Self-maintaining and instrumental activities of daily living. Gerontologist.

[19] Hilgenkamp T.I.M., van Wijck R., Evenhuis H.M. (2011). (Instrumental) activities of daily living in older adults with intellectual disabilities. Res. Dev. Disabil..

[20] Peterson M.D., Ryan J.M., Hurvitz E.A., Mahmoudi E. (2015). Chronic conditions in adults with cerebral palsy. JAMA.

[21] Majnemer A., Shevell M., Hall N., Poulin C., Law M. (2010). Developmental and functional abilities in children with cerebral palsy as related to pattern and level of motor function. J. Child. Neurol..

[22] Strax T.E., Luciano L., Dunn A.M., Quevedo J.P. (2010). Aging and developmental disability. Phys. Med. Rehabil. Clin. N. Am..

[23] Jopp D.A., Keys C.B. (2001). Diagnostic overshadowing reviewed and reconsidered. Am. J. Ment. Retard..

[24] Krahn G.L., Fox M.H. (2014). Health disparities of people with intellectual disabilities: What do we know and what can we do?. J. Appl. Res. Intellect. Disabil..

[25] McCarron M., Haigh M., McCallion P. (2017). Health, Wellbeing and Social Inclusion: Ageing with an Intellectual Disability in Ireland.

[26] Griffith G.M., Hastings R.P. (2014). ‘He’s hard work, but he’s worth it.’ The experience of caregivers of individuals with intellectual disabilities and challenging behaviour: A meta-synthesis of qualitative research. J. Appl. Res. Intellect. Disabil..

[27] Murthy S., Parker Harris S., Hsieh K. (2024). Formal support and service needs of family caregivers of adults with intellectual and/or developmental disabilities in India. J. Appl. Res. Intellect. Disabil..

[28] Strydom A., Shooshtari S., Lee L., Raykar V., Torr J., Tsiouris J., Jokinen N., Courtenay K., Bass N., Sinnema M. (2010). Dementia in older adults with intellectual disabilities—Epidemiology Presentation, and Diagnosis. J. Policy Pract. Intellect. Disabil..

[29] Haak P., Majnemer A., Rosenbaum P., Abrahamowicz M. (2009). Development of functional abilities in children with cerebral palsy. Dev. Med. Child. Neurol..

[30] Halfon N., Hochstein M. (2002). Life course health development: An integrated framework for developing health, policy, and research. Milbank Q..

[31] Korea Institute for Health and Social Affairs, Seoul National University (2023). User’s Guide for the 18th Wave of the Korean Welfare Panel Study.

[32] Morris J.F., Temple W. (1985). Spirometric “lung age” estimation for motivating smoking cessation. Prev. Med..

[33] Cole J.H., Franke K. (2017). Predicting age using neuroimaging: Innovative brain ageing biomarkers. Trends Neurosci..

[34] Bittles A.H., Glasson E.J. (2004). Clinical, social, and ethical implications of changing life expectancy in Down syndrome. Dev. Med. Child Neurol..

[35] Silverman W.P., Zigman W.B., Krinsky-McHale S.J., Ryan R., Schupf N. (2013). Intellectual disability, mild cognitive impairment, and risk for dementia. J. Policy Pract. Intellect. Disabil..

[36] Bigby C., Frawley P. (2020). A new paradigm for the social inclusion of people with developmental disability across the life course. Social Inclusion and People with Developmental Disability.

[37] Emerson E., Hatton C. (2007). The socio-economic circumstances of children at risk of disability in Britain. Disabil. Soc..

[38] Wang S., Phillips D., Lee J. (2021). Disability prevalence in midlife (aged 55–65 years): Cross-country comparisons of gender differences and time trends. Womens Midlife Health.

[39] Sullivan W.F., Berg J.M., Bradley E., Cheetham T., Denton R., Heng J., Hennen B., Joyce D., Kelly M., Korossy M. (2011). Primary care of adults with developmental disabilities: Canadian consensus guidelines. Can. Fam. Physician.

[40] Ali A., Scior K., Ratti V., Strydom A., King M., Hassiotis A. (2013). Discrimination and other barriers to accessing health care: Perspectives of patients with mild and moderate intellectual disability and their carers. PLoS ONE.

[41] Braddock D.L., Hemp R.E., Tanis E.S., Wu J., Haffer L. (2017). The State of the States in Intellectual and Developmental Disabilities.

